# Altered B-lymphopoiesis in mice with deregulated thrombopoietin signaling

**DOI:** 10.1038/s41598-017-15023-2

**Published:** 2017-11-02

**Authors:** Amanda E. Au, Marion Lebois, Starling A. Sim, Ping Cannon, Jason Corbin, Pradnya Gangatirkar, Craig D. Hyland, Diane Moujalled, Angelika Rutgersson, Fatme Yassinson, Benjamin T. Kile, Kylie D. Mason, Ashley P. Ng, Warren S. Alexander, Emma C. Josefsson

**Affiliations:** 1 The Walter and Eliza Hall Institute of Medical Research, 1 G Royal Parade, VIC 3052 Australia; 2 The University of Melbourne, Department of Medical Biology, 1 G Royal Parade, VIC 3052 Australia; 3 Department of Pharmacology, Institute of Neuroscience and Physiology, Sahlgrenska Academy, University of Gothenburg, P.O. Box 431, 405 30 Gothenburg, Sweden; 4 The University of Melbourne, Faculty of Medicine, Dentistry and Health Sciences, Melbourne, VIC Australia; 5 Present Address: Monash Biomedicine Discovery Institute, Department of Anatomy and Developmental Biology, Monash University, Clayton, VIC 3800 Australia

## Abstract

Thrombopoietin (TPO) is the master cytokine regulator of megakaryopoiesis. In addition to regulation of megakaryocyte and platelet number, TPO is important for maintaining proper hematopoietic stem cell (HSC) function. It was previously shown that a number of lymphoid genes were upregulated in HSCs from *Tpo* ^−/−^ mice. We investigated if absent or enhanced TPO signaling would influence normal B-lymphopoiesis. Absent TPO signaling in *Mpl* ^−/−^ mice led to enrichment of a common lymphoid progenitor (CLP) signature in multipotential lineage-negative Sca-1^+^c-Kit^+^ (LSK) cells and an increase in CLP formation. Moreover, *Mpl* ^−/−^ mice exhibited increased numbers of PreB2 and immature B-cells in bone marrow and spleen, with an increased proportion of B-lymphoid cells in the G1 phase of the cell cycle. Conversely, elevated TPO signaling in *Tpo* ^*Tg*^ mice was associated with reduced B-lymphopoiesis. Although at steady state, peripheral blood lymphocyte counts were normal in both models, *Mpl* ^−/−^ Eµ-*myc* mice showed an enhanced preneoplastic phase with increased numbers of splenic PreB2 and immature B-cells, a reduced quiescent fraction, and augmented blood lymphocyte counts. Thus, although Mpl is not expressed on lymphoid cells, TPO signaling may indirectly influence B-lymphopoiesis and the preneoplastic state in *Myc*-driven B-cell lymphomagenesis by lineage priming in multipotential progenitor cells.

## Introduction

Platelets are produced from large precursor cells in the bone marrow called megakaryocytes. The process by which megakaryocytes produce platelets is unique and involves polyploidisation and shedding of platelets into the blood stream^1^. Thrombopoietin (TPO), produced mainly in the liver, stimulates both megakaryocyte colony formation and enhances megakaryocyte maturation in the bone marrow leading to increased platelet production. TPO binds to its receptor, TPOR/MPL, which is expressed on hematopoietic stem cells (HSCs), megakaryocytes and platelets^2^. Circulating TPO concentrations are controlled by MPL via receptor-mediated internalization and degradation^3,4^, and in part by sensing of senescent platelets by the Ashwell-Morell receptor in the liver^5^. *Mpl* ^−/−^ mice exhibit platelet counts ~10% of wild-type levels^6^, while *Tpo* ^*Tg*^ transgenic mice, engineered to overexpress TPO in the liver, have ~3.5 times higher platelet counts^7^ compared to wild-type mice. In addition to regulating megakaryopoiesis, TPO is known to affect HSC number and quiescence^7^. A low level of TPO leads to reductions in total HSCs, but with more of them in active cycle. This is evident in *Mpl* deficient mice, which not only exhibit thrombocytopenia, but also have increased cycling and a decline in the number of HSCs with age^8–10^. Conversely, a high TPO level leads to a greater proportion of quiescent HSCs^7^.

It has previously been reported that down-regulation of Mpl marks the transition from pluripotent to lymphoid-primed multipotent hematopoietic progenitor cells^11^ and that a number of lymphoid genes are upregulated in HSCs from *Tpo* ^−/−^ mice^12^. Here, we compared the effects of absent or enhanced TPO signaling on steady-state B-lymphopoiesis and in a model of over-active B-lymphopoiesis, the Eµ-*myc* transgenic mice, which display a characteristic preneoplastic phase with expansion of immature Pro- and Pre-B cell populations^13,14^. We found that, although Mpl is not expressed on lymphoid cells, absent TPO signaling in *Mpl* ^−/−^ mice led to lineage priming of lineage-negative Sca-1^+^c-Kit^+^ (LSK) cells with increased numbers of common lymphoid progenitors (CLPs), PreB2 and immature B-cells. Additionally, *Mpl* ^−/−^ Eµ-*myc* mice demonstrated an enhanced preneoplastic phase that resulted in earlier disease onset. By comparison, elevated TPO signaling modestly suppressed B-lymphopoiesis and resulted in delayed disease onset in *Tpo* ^*Tg*^ Eµ-*myc* transgenic mice. Thus, despite the absence of Mpl expression in B-lymphoid cells, changes induced in multipotential hematopoietic progenitor cells by altered TPO signaling, including altered gene expression and lineage priming, indirectly affect B-lymphopoiesis. The effects of altered Mpl signaling at steady-state were confined to changes in B-lymphoid precursor cell numbers, with no alteration in circulating B cell number. However, under conditions of excess proliferation modeled using Eµ-*myc* transgenic mice, the changes conferred by altered Mpl signaling in preneoplastic B-lymphopoiesis were sufficient to influence the onset of B-cell lymphoma.

## Results

### Lymphoid lineage skewing in Mpl^−/−^ bone marrow and spleen

**Figure 1:**
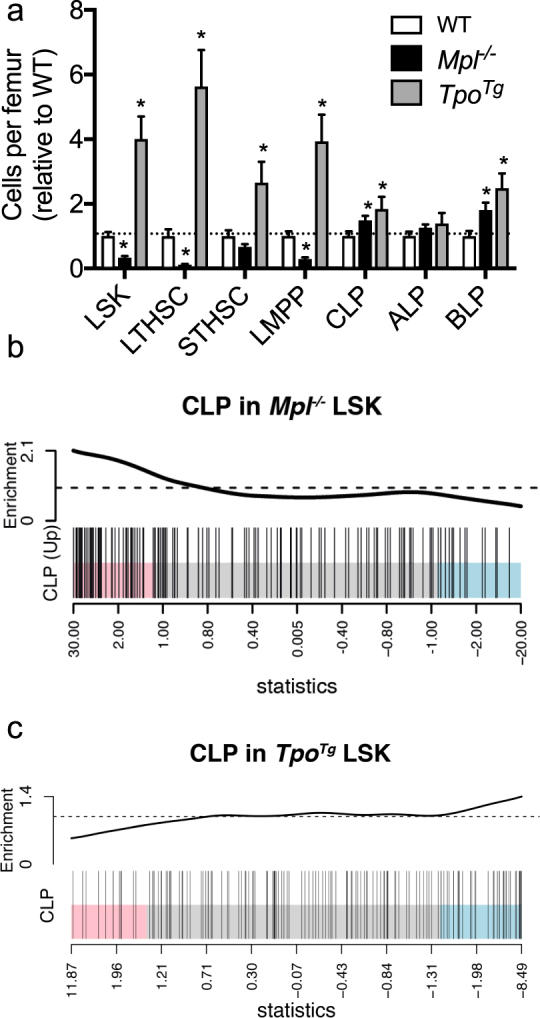
Lymphoid lineage priming in *Mpl* ^−/−^ LSKs. (**a**) Hematopoietic stem and progenitor cells per femur relative to WT in 4–5 weeks old mice. WT (n = 8), *Mpl* ^−/−^ (n = 6) and *Tpo* ^*Tg*^ (n = 7). Mean ± SEM. Statistical significance was generated by Student’s unpaired t-test. *p < 0.05. (**b**) Barcode plot of the CLP gene signature in *Mpl* ^−/−^ and (**c**) *Tpo* ^*Tg*^ LSKs. Gene set tests were performed using gene set obtained for the CLP population from the Hemopedia atlas^18^.

In accordance with previous findings reported in adult mice^7,15^, in the setting of increased TPO signaling, we found that 4–5 week old *Tpo* ^*Tg*^ mice had a 4-fold increase in numbers of LSK cells and corresponding LSK subpopulations long-term HSCs (LTHSCs), short-term HSCs (STHSCs) and lymphoid-primed multipotent progenitors (LMPPs) in the bone marrow when compared to wild-type mice (Fig. 1a). While augmented numbers of CLPs and B-cell primed progenitors (BLPs), could also be observed in *Tpo* ^*Tg*^ bone marrow, this effect was more modest (Fig. 1a, Supplementary Fig. S1). In contrast, as previously described^7,15^, *Mpl* ^−/−^ mice had a significantly reduced number of LSKs (Fig. 1a, Supplementary Fig. S1). Interestingly, despite a deficiency in the number of LSKs, numbers of CLPs and BLPs were significantly increased in *Mpl* ^−/−^ bone marrow (Fig. 1a, Supplementary Fig. S1). Prior evidence had suggested that lymphoid-specific gene transcription may be primed early in hematopoiesis^16,17^. Here, we observed more specifically, that relative to wild-type LSKs, a CLP gene expression signature^18^ was upregulated in *Mpl* ^−/−^ LSKs (*p* = 0.0001) (Fig. 1b, Supplementary Table S1) and conversely down regulated in *Tpo* ^*Tg*^ LSKs (*p* = 0.0001) (Fig. 1c, Supplementary Table S1). In confirmation of these results, lineage priming with early B-cell progenitor ProB and PreB gene expression signatures was also observed in *Mpl* ^−/−^ LSKs, and that these were conversely downregulated in *Tpo* ^*Tg*^ LSKs (Supplementary Table S2).

**Figure 2:**
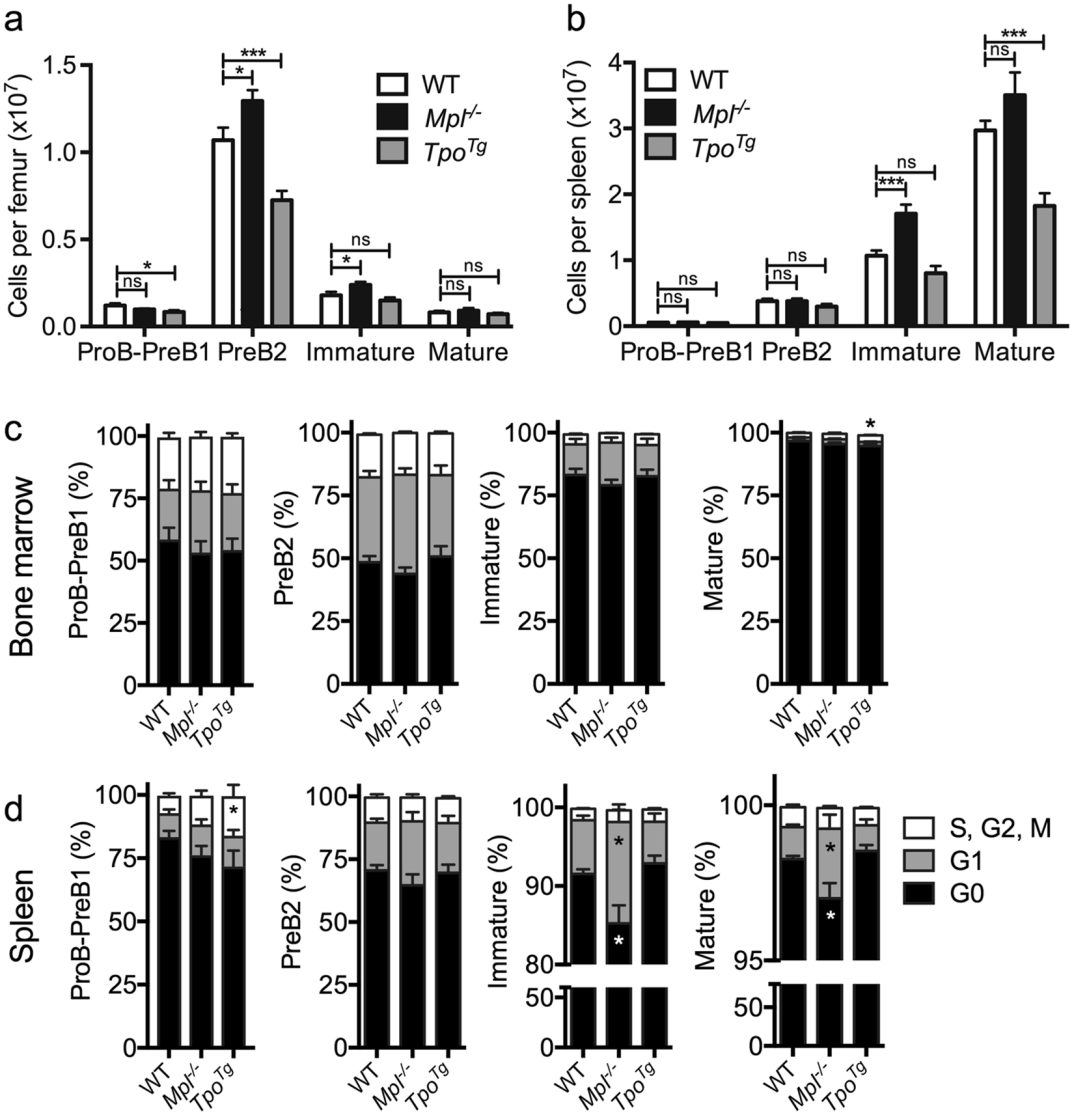
Lymphoid lineage priming in *Mpl* ^−/−^ bone marrow and spleen. (**a**) Bone marrow and (**b**) spleen cellularity of B-cell subsets in 4–5 week old mice. WT (n = 17), *Mpl* ^−/−^ (n = 16) and *Tpo* ^*Tg*^ (n = 15). (**c-d**) K_i_-67/DAPI cell cycling assessment of B-cell subsets within bone marrow and spleen of 4–5 week old WT (n = 17), *Mpl* ^−/−^ (n = 17) and *Tpo* ^*Tg*^ (n = 13) mice. Statistical significance was generated by Student’s unpaired t-test. Mean ± SEM. *p < 0.05; **p < 0.005; ***p < 0.001

To determine any functional consequences of these altered gene expression profiles, the numbers of lymphoid cells were further enumerated in *Mpl* ^−/−^ and *Tpo* ^*Tg*^ mice. In 4–5 week old mice, *Mpl* deficiency led to increased PreB2 cell numbers in bone marrow along with elevated immature B-cell numbers in bone marrow and spleen (Fig. 2a,b). In contrast, *Tpo* ^*Tg*^ mice had reduced numbers of bone marrow ProB-PreB1, PreB2 cells and splenic mature B cells (Fig. 2a,b).

While Mpl is expressed on LSK cells, we found the receptor to be absent on CLPs, ProB-PreB1, PreB2, immature, and mature B cells (Supplementary Fig. S1), excluding an effect via direct TPO signaling in committed B-lineage cells. Limiting dilution assays revealed no significant difference in *in vitro* clonogenicity of purified *Mpl* ^−/−^ and *Tpo* ^*Tg*^ CLPs relative to wild-type cells (Supplementary Fig. S2). However, in the *Mpl* ^−/−^ spleen, immature and mature B-cells cycled significantly more than wild-type, as indicated by an increased proportion of cells in G1 and a reduced proportion in G0 (Fig. 2d). However, changes in cell cycle status were not evident in B-lymphoid cells in *Tpo* ^*Tg*^ mice (Fig. 2c,d), nor in *Mpl* ^−/−^ bone marrow (Fig. 2c), and peripheral blood lymphocyte counts in *Mpl* ^−/−^ and *Tpo* ^*Tg*^ mice were normal (Table 1)^15^. Thus, although altered TPO signaling may indirectly affect B-lymphopoiesis, other mechanisms were clearly contributing to circulating lymphocyte homeostasis at steady state.

### TPO restricts Eµ-myc preneoplastic lymphoproliferation

**Figure 3:**
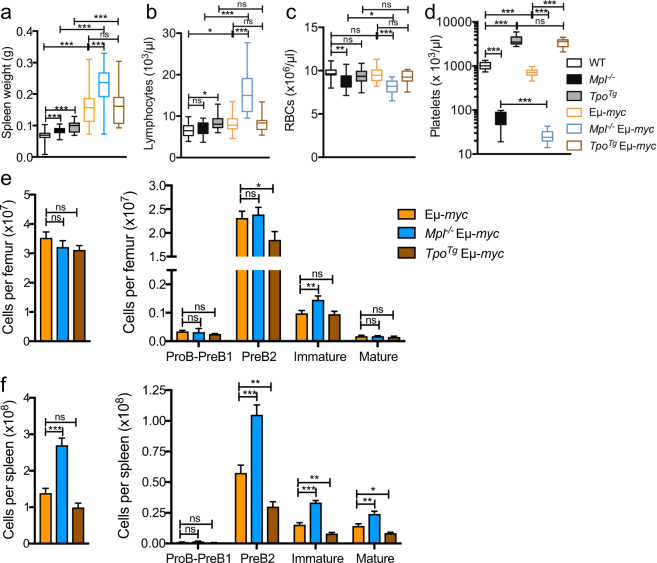
Increased lymphoproliferation in preneoplastic *Mpl* deficient mice. (**a**) Spleen weight (**b**) Lymphocyte (**c**) Red blood cell (RBC) and (**d**) platelet counts in 4–5 week old mice. n = 11–18 mice per genotype. Whiskers: Min to Max. (**e**) Bone marrow and (**f**) spleen cellularity and Pro-PreB1, PreB2, immature and mature B-cells in 4–5 week old mice assessed by flow cytometry. Data are presented as mean ± SEM. n = 11–18 mice per genotype. Statistical significance was generated by Student’s unpaired t-test. *p < 0.05; **p < 0.005; ***p < 0.001

**Figure 4:**
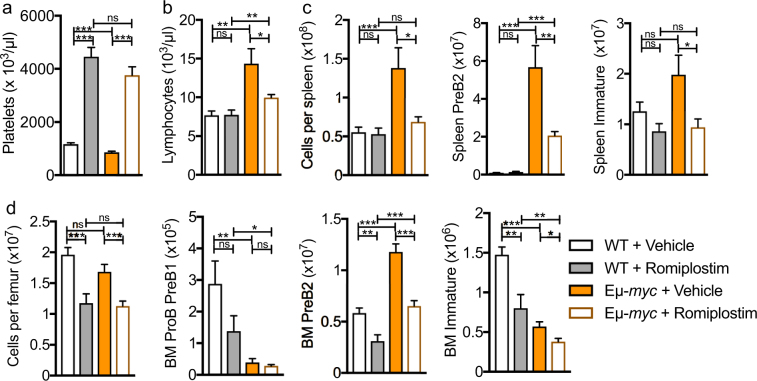
Effects of TPO-mimetic on preneoplastic phase. 1-week-old Eμ*-myc* mice and their wild-type littermates were injected subcutaneously every 3 days with 100 μg/kg Romiplostim or saline vehicle. At 5 weeks of age (**a**) platelet and (**b**) lymphocyte counts (**c**) spleen cellularity, PreB2, and immature B-cells, and (**d**) bone marrow cellularity, ProB PreB1, PreB2 and Immature B-cells. n = 8–14 mice per group. Data are presented as mean ± SEM. Statistical significance was generated by Student’s unpaired t-test. *p < 0.05; **p < 0.005; ***p < 0.001. BM = bone marrow.

We next investigated the changes in B-lymphopoiesis evident in *Mpl* ^−/−^ and *Tpo* ^*Tg*^ mice in the context of excess B-lymphoid proliferation in Eµ-*myc* transgenic mice^14^. We initially confirmed that preneoplastic Eµ-*myc* B-cell subsets did not express Mpl protein, when assessed by flow cytometry (Supplementary Fig. S3) as was the case for wild-type B-cell subsets. The preneoplastic phase in Eµ-*myc* mice is characterized by a polyclonal expansion of PreB cells accompanied by mild splenomegaly^19^. We observed that relative to 4–5 week-old Eµ-*myc* control mice, age matched *Mpl* ^−/−^ Eµ-*myc* mice displayed an increase in spleen weight of 1.4-fold (Fig. 3a) which was accompanied by a 1.9-fold increase in peripheral blood lymphocyte counts (Fig. 3b). In comparison to Eµ-*myc* controls, *Mpl* ^−/−^ Eµ-*myc* mice showed a modest reduction in red blood cell number, while, the number of platelets was reduced in the presence of the Eµ-*myc* transgene irrespective of whether mice were on a wild-type or *Mpl* ^−/−^ background (Fig. 3c,d). In order to investigate if the observed blood lymphocyte elevation corresponded with changes in B cell precursors in the bone marrow and spleen we examined B-cell subsets and cellularity in pre-neoplastic Eµ-*myc*, *Mpl* ^−/−^ Eµ-*myc* and *Tpo* ^*Tg*^ Eµ*-myc* mice (Fig. 3e,f). We found increased spleen cellularity and a doubling in the number of PreB2 cells as well as significant increases in the numbers of immature and mature B-cells in pre-neoplastic *Mpl* ^−/−^ Eµ*-myc* spleen, compared to Eµ-*myc* mice (Fig. 3f). Moreover, *Mpl* ^−/−^ Eµ*-myc* mice had increased numbers of immature B-cells in the bone marrow (Fig. 3e). Conversely, pre-neoplastic *Tpo* ^*Tg*^ Eµ*-myc* mice had reduced PreB2 cell numbers in bone marrow and spleen as well as significantly fewer immature and mature splenic B-cells compared to Eµ*-myc* control mice (Fig. 3e,f). To confirm these results, we next assessed the effect of exogenous TPO on the preneoplastic phase by administration of the TPO-mimetic Romiplostim^20^ in Eµ-*myc* and littermate control animals starting when they were 1 week old. When mice were assessed at 5-weeks of age, platelet counts were significantly increased in animals receiving Romiplostim compared to vehicle (Fig. 4a). As expected, 5-week old vehicle treated Eµ-*myc* mice had increased lymphocyte counts, spleen cellularity and bone marrow and spleen PreB2 cells compared to wild-type control mice (Fig. 4b,c,d). Interestingly, Romiplostim treated Eµ-*myc* mice had reduced lymphocyte counts, lower spleen cellularity and fewer PreB2 and immature B-cells in both bone marrow and spleen when compared to vehicle treated Eµ-*myc* mice (Fig. 4b,c,d).

**Figure 5:**
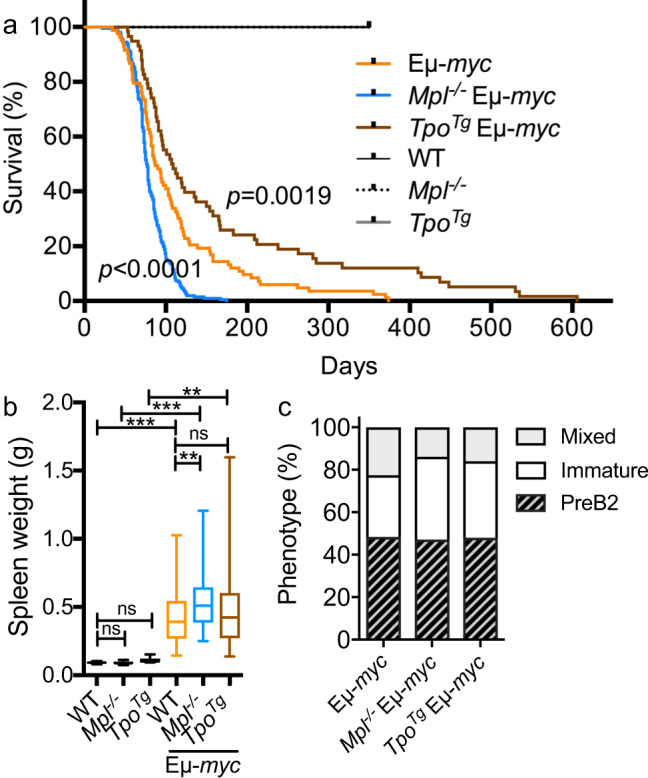
TPO delays B-cell lymphoma development in Eµ-*myc* mice. (**a**) Survival of Eµ-*myc* (n = 83), *Mpl* ^−/−^ Eµ-*myc* (n = 200), *Tpo* ^*Tg*^ Eµ-*myc* (n = 58), WT (n = 14), *Mpl* ^−/−^ (n = 11) and *Tpo* ^*Tg*^ (n = 13) mice. *P-*values were generated with Logrank (Mantel-Cox) test. (**b**) Spleen weight in terminally ill mice and 1-year old control mice. Eµ-*myc* (n = 47), *Mpl* ^−/−^ Eµ-*myc* (n = 59), *Tpo* ^*Tg*^ Eµ-*myc* (n = 56), WT (n = 5), *Mpl* ^−/−^ (n = 7) and *Tpo* ^*Tg*^ (n = 6). Whiskers: Min to Max. Statistical significance was generated by Student’s unpaired t-test. *p < 0.05; **p < 0.005; ***p < 0.001. (**c**) Bone marrow disease phenotype in terminally ill mice assessed by flow cytometry. Eµ-*myc* (n = 31), *Mpl* ^−/−^ Eµ-*myc* (n = 36) and *Tpo* ^*Tg*^ Eµ-*myc* (n = 25) mice.

Eµ-*myc* mice have previously been shown to succumb to aggressive lymphoma within 3–6 months after birth^14^. *Mpl* ^−/−^, *Tpo* ^*Tg*^ and wild-type mice aged up to 1 year showed no signs of tumor development during the course of the study (Fig. 5a). However, the changes conferred by altered Mpl signaling in preneoplastic B-lymphopoiesis were sufficient to influence the onset of B cell lymphoma in Eµ*-myc* mice. *Mpl* ^−/−^ Eµ*-myc* mice became unwell with a reduced median survival of 76.5 days, compared to conventional Eµ*-myc* mice (87 days, *p* < 0.0001) (Fig. 5a). Conversely, *Tpo* ^*Tg*^ Eµ*-myc* mice exhibited delayed illness (*p* = 0.0019) with a median survival of 107 days (Fig. 5a). Moribund mice of all three genotypes had splenomegaly (Fig. 5b) and many presented with elevated blood lymphocyte counts (Table 1) as well as enlarged lymph nodes (Supplementary Fig. 4b). Spleen weights and cellularity were significantly increased in ill *Mpl* ^−/−^ Eµ*-myc* mice when compared to control Eµ*-myc* mice (Fig. 5b, Supplementary Fig. S4a). The bone marrow revealed diffuse tumor infiltrate of large cells with multiple nucleoli, as well as frequent extravasation into surrounding tissues (Supplementary Fig. S5). The tumor phenotypes, classified as PreB2, immature B-cell or a mixture of the two (mixed), were similarly distributed among the genotypes bearing the Eµ-*myc* transgene (Fig. 5c). Hematocrit and platelet counts often drop in the late stages of hematological malignancy including in Eµ*-myc* induced lymphoma (Table 1). Of note, *Mpl* ^−/−^ Eµ-*myc* mice were substantially more anemic than Eµ*-myc* and *Tpo* ^*Tg*^ Eµ*-myc* mice (Table 1). At necropsy, lymph node hemorrhage was often observed in *Mpl* ^−/−^ Eµ*-myc* mice, a phenotype not commonly present in the other genotypes (Supplementary Fig. S6).

**Table 1:** Peripheral blood counts from ~1 year-old mice (WT, *Mpl* ^-/-^ and *Tpo* ^Tg^) and terminally ill Eµ*-myc* mice.

	WT	*Mpl* ^−/−^	*Tpo* ^*Tg*^	Eµ*-myc*	*Mpl* ^−/−^ Eµ*-myc*	*Tpo* ^*Tg*^ Eµ*-myc*
	n = 14	n = 11	n = 13	n = 58	n = 81	n = 62
Platelets (x 10^6^/ml)	1338 ± 245	172 ± 145^***^	4748 ± 745^***^	704 ± 283^***^	31 ± 16^***,^^^^	2227 ± 951^***,^^^^
MPV (femtoliter)	7.5 ± 1.3	10.9 ± 4.1^**^	8.4 ± 1.2	7.3 ± 1.4	11.8 ± 3.2^***,^^^^	7.3 ± 1.2
Hematocrit (%)	55.0 ± 3.3	49.3 ± 8.9	52.0 ± 3.5	49.2 ± 8.5^*^	33.6 ± 10.5^***,^^^^	47.3 ± 4.6^**^
RBCs (x10^9^/ml)	10.5 ± 0.7	9.6 ± 1.8	9.8 ± 1.4	9.7 ± 1.7	6.2 ± 2.1^***,^^^^	9.8 ± 0.9
Leukocytes (x10^6^/ml)	8.1 ± 3.2	6.9 ± 2.7	10.2 ± 3.5	57.4 ± 77	35.9 ± 36	87.2 ± 101^***^
Lymphocytes (x10^6^/ml)	6.3 ± 2.4	5.3 ± 2.2	8.2 ± 3.3	27.7 ± 38	23.0 ± 23	49.5 ± 60^***,^^^
Neutrophils (x10^6^/ml)	1.2 ± 0.4	0.8 ± 0.3	1.6 ± 0.6	5.7 ± 4.2^**^	3.0 ± 2.6^^^^^	9.9 ± 6.0^***,^^^^
Monocytes (x10^6^/ml)	0.1 ± 0.0	0.1 ± 0.1	0.2 ± 0.1	0.2 ± 0.3	0.2 ± 0.3	0.7 ± 0.5^***,^^^^
Eosinophils (x10^6^/ml)	0.3 ± 0.4	0.1 ± 0.1	0.2 ± 0.1	0.2 ± 0.2	0.2 ± 0.2	0.4 ± 0.3^^^^

Data represent mean ± SD, 1 way ANOVA with Dunnett’s multiple comparison test. Significance compared to wild-type (WT) *p < 0.05; **p < 0.005; ***p < 0.001; significance compared to Eµ-*myc* ^^*p* < 0.005; ^^^*p* < 0.001. Mean platelet volume (MPV), red blood cell (RBCs).

### Enhanced cycling of B-lymphoid cells in pre-neoplastic Mpl^−/−^ Eµ-myc mice

Our results support a model in which the pre-malignant phase of Eµ*-myc* lymphomagenesis is affected by the underlying cellular changes in mice with altered TPO signaling. Since increased B-lymphoid proliferation in pre-malignant Eµ*-myc* mice is characterized by changes in apoptosis and cell cycling^19,21^, we examined these processes in the compound mutant mice. Bone marrow cells from 4–5 week old Eµ*-myc*, *Mpl* ^−/−^ Eµ*-myc*, and *Tpo* ^*T*g^ Eµ*-myc* mice were cultured for 24 hours under conditions of cytokine deprivation, and apoptosis was measured in B-cell subsets by Annexin-V binding. In accord with previous studies^21^, Eµ-*myc* B-cell subsets were highly sensitive to apoptosis compared to wild-type control cells (Supplementary Fig. S7). However, no differences in B-cell apoptosis were observed in *Mpl* ^−/−^ Eµ*-myc* or *Tpo* ^*T*g^ Eµ*-myc* cells (Supplementary Fig. S7).

**Figure 6:**
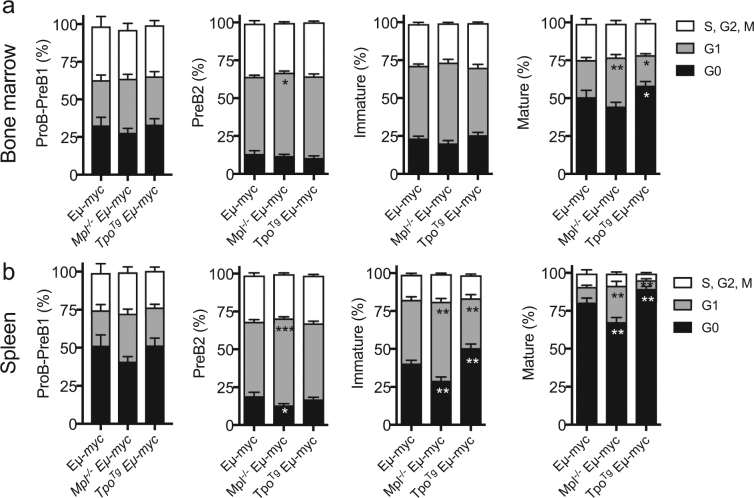
Increased B-cell cycling in preneoplastic *Mpl* ^−/−^ Eµ-*myc* mice. Cell cycle status of (**a**) bone marrow and (**b**) spleen B-cell subsets in 4–5 week old Eµ-*myc* (n = 13), *Mpl* ^−/−^ Eµ-*myc* (n = 22) and *Tpo* ^*Tg*^ Eµ-*myc* (n = 13) mice assessed by K_i_-67/DAPI staining profiles. Statistical significance was compared to Eµ-*myc*. Statistical significance was generated by Student’s unpaired t-test. Mean ± SEM. *p < 0.05; **p < 0.005; ***p < 0.001.

TPO has an established role in the control of HSC number and quiescence^6,7^, and we confirmed reduced quiescence (G0) in *Mpl* ^−/−^ LSKs and conversely increased quiescence in *Tpo* ^*T*g^ LSKs (Supplementary Fig. S8c). Eµ-*myc* LSKs expressed Mpl to the same extent as wild-type cells (Supplementary Fig. S8a) and similar changes in LSK cell number and cell cycling were observed in *Mpl* ^−/−^ and *Tpo* ^*T*g^ mice in the presence and absence of Eµ-*myc* (Supplementary Fig. S8b,c). We next investigated if there were differences in cycling of ProB-PreB1, PreB2, immature and mature B-cells in bone marrow and spleen of preneoplastic *Mpl* ^−/−^ Eµ*-myc*, *Tpo* ^*T*g^ Eµ-*myc*, and Eµ*-myc* mice. Previous studies have shown that Eµ*-myc* mice have more than double the number of PreB cells and mature B cells in active cycle compared with controls^19^. In keeping with these findings we found an increased proportion of PreB2, immature and mature B-cells in active cycle in Eµ-*myc* mice compared to healthy controls (Figs 2 and 6). Consistent with our findings in *Mpl* ^−/−^ mice, this effect was augmented in *Mpl* ^−/−^ Eµ*-myc* PreB2-, immature and mature B-lymphocytes, while a reverse effect was observed in immature and mature B-cells in *Tpo* ^*T*g^ Eµ*-myc* mice, particularly in the spleen (Fig. 6a,b). Thus, the accumulation of B-lymphoid cells in preneoplastic *Mpl* ^−/−^ Eµ-*myc* mice is likely to be the result of further deregulation of the already increased rate of proliferation of Eµ-*myc* B-lymphoid cells, with a converse attenuating effect of excess TPO signaling in *Tpo* ^*T*g^ Eµ*-myc* mice.

## Discussion

Our findings demonstrated that perturbed TPO signaling had a clear effect on the lymphoid compartment at steady state using genetically modified *Mpl* ^−/−^ and *Tpo* ^*Tg*^ models. The earliest stages of the hematopoietic hierarchy where this effect was apparent was observed in Mpl expressing LSK cells, where an upregulation of CLP, ProB and PreB gene expression signatures in *Mpl* ^−/−^ LSK cells confirmed prior data from Sanjuan-Pla *et al*. that showed an upregulation of lymphoid genes in HSCs from *Tpo* ^−/−^ mice, while *Tpo* ^−/−^ bone marrow cells gave lymphoid-biased reconstitution when transplanted^12^. Together, these data suggest absence of TPO signaling on LSK cells results in lineage priming with enhanced early lymphoid gene expression. In support of the biological significance of the upregulated CLP, ProB and PreB signatures in *Mpl* ^−/−^ LSKs, we found an increased number of CLPs in *Mpl* ^−/−^ bone marrow. Moreover, we demonstrate that 4–5 week old *Mpl* ^−/−^ mice exhibit increased PreB2 and immature B-cell counts in bone marrow and spleen, with an increased proportion of immature and mature splenic B-cells in active cycle. Conversely, elevated TPO signaling in *Tpo* ^*Tg*^ mice negatively affected bone marrow and spleen B-lymphopoiesis. These effects on committed B-lymphoid precursor cell numbers must be indirect, since previous studies, also confirmed here, have shown that while LSK cells express the Mpl receptor, CLP and B-cell subsets do not^11,15,22–24^. The *in vitro* clonogenicity of CLPs from *Mpl* ^−/−^ and *Tpo* ^*Tg*^ mice was unaltered, but changes in the cell cycling profiles of B-lymphoid precursor cells correlated with altered cell numbers in these mice. Interestingly, the master erythroid cytokine regulator, EPO, has also been shown, in addition to stimulating erythropoiesis, to negatively affect B-lymphopoiesis at the ProB-cell and PreB-cell stage of maturation, by an indirect mechanism within the bone marrow environment^25^.

The altered B-lymphopoiesis observed in *Mpl* ^−/−^ and *Tpo* ^*Tg*^ bone marrow and spleen was, however, not sufficient to influence lymphocyte counts in the peripheral blood at steady state. Nevertheless, we hypothesized that changes to B-lymphopoiesis evident in mice with altered TPO signaling might have more dramatic consequences in a perturbed system. Thus, we investigated the preneoplastic phase in Eµ-*myc* transgenic mice, which is characterized by increased B-lymphoid cell cycling and lymphocytosis. We observed accumulation of PreB2, immature and mature B-cells in preneoplastic *Mpl* ^−/−^ Eµ-*myc* mice relative to that in Eµ-*myc* controls, an effect that was reversed in *Tpo* ^*Tg*^ Eµ-*myc* mice. Furthermore, the effect on preneoplastic Eµ-*myc* mice was also countered by 4 weeks of Romiplostim administration, when mice were 1 to 5 weeks of age. While the increased apoptosis characteristic of Eµ-*myc* B-lymphoid cells^21^ was unaltered in *Mpl* ^−/−^ Eµ-*myc* and *Tpo* ^*Tg*^ Eµ-*myc* mice, the increased B-lymphoid cell cycling caused by Eµ-*myc* expression was augmented in *Mpl* ^−/−^ Eµ-*myc* mice and attenuated in *Tpo* ^*Tg*^ Eµ-*myc* animals. The effects on the preneoplastic phase in Eµ-*myc* mice were sufficient to influence disease outcome: illness from B-lymphoma developed with a reduced latency in *Mpl* ^−/−^ Eµ-*myc* mice and was delayed in *Tpo* ^*Tg*^ Eµ-*myc* mice. This result is in agreement with a recent study which reported that *Mpl* deficiency increased precursor B-ALL development in the *Lnk* ^−/−^ *Tp53* ^−/−^ mouse model^26^.

Our results support a model in which the direct influence of the Eµ-*myc* transgene and the indirect effect of loss of TPO signaling, at least in part due to lymphoid lineage priming in multipotential hematopoietic cells, appear to have an additive effect on cell cycling and accumulation of B-lymphoid populations resulting in an enhanced preneoplastic state and earlier disease onset in *Mpl* ^−/−^ Eµ-*myc* mice. The suppressive indirect impact of excess TPO signaling in *Tpo* ^*Tg*^ mice was also evident in the presence of the Eµ-*myc* transgene and could account for the delayed disease initiation in *Tpo* ^*T*g^ Eµ*-myc* mice. Lymph node intratumor hemorrhage was occasionally observed in terminally ill *Mpl* ^−/−^ Eµ-*myc* mice, which is consistent with previous recognition of hemorrhaging of solid tumors in severely thrombocytopenic mice^27,28^. Thus, while excess bleeding may have contributed to the terminal stage of disease in *Mpl* ^−/−^ Eµ*-myc* mice, the significant preneoplastic expansion of splenic B lymphoid cell numbers (PreB2, immature and mature B-cells) and blood lymphocyte counts support a significant contribution by altered B-lymphopoiesis.

While no megakaryocyte-associated fibrosis^29,30^ was observed in 4–5 week old *Tpo* ^*Tg*^ mice (data not shown), our data cannot exclude an effect on B-lymphopoiesis from altered environmental niches or competition for space arising from abnormal megakaryocyte number in the bone marrow and spleen as *Mpl* ^−/−^ mice have 10% of normal megakaryocyte and platelet numbers, while *Tpo* ^*Tg*^ mice have ~3.5-fold increased platelet counts with 3-fold elevated bone marrow and spleen megakaryocytes^7,15,31^ (Supplementary Fig. S5b).

In addition, platelets have been shown to protect tumor vasculature^28^ and to promote hematogenous metastasis in mouse models^32,33^. Although limited information is available on roles of platelets in lymphoma and leukemia, some recent studies have examined interactions between lymphoma and leukemia cell lines and activated platelets or platelet-released molecules^34–36^. Although, the present study investigated the preneoplastic phase in Eµ-*myc* mice, future studies are needed to explore potential effects of altered megakaryocyte or platelet numbers that may alter the biological behavior of established lymphoma.

## Material and Methods

### Mice

Eµ*-myc* ^14^, *Mpl* ^−/−^^6^ and *Tpo* ^*Tg*^ ^7^ mice have been previously described. All mutations were backcrossed onto the C57BL/6 background for at least 10 generations prior to this study. Experiments include balanced groups of male and female mice if not otherwise stated. Eµ*-myc* mice included in the survival study were taken at an ethical endpoint when displaying lymphadenopathy and/or splenomegaly. All animal experiments complied with the regulatory standards of, and were approved by the Walter and Eliza Hall Institute (WEHI) Animal Ethics Committee.

### Peripheral blood counts

Automated cell counts were performed on blood collected from the retro-orbital plexus or by cardiac puncture into Microtainer tubes containing EDTA (Sarstedt, Ingle Farm, SA Australia), using an Advia 2120 hematological analyser (Siemens, Munich Germany).

### Progenitor and B-cell subset analysis

4–5 week old, 1–year old or terminally ill mice were analyzed. Organ processing: Bone marrow was flushed from femurs and tibias in 10 mL of balanced salt solution (BSS; 150 mM NaCl, 3.7 mM KCl, 2.5 mM CaCl_2_, 1.2 mM MgSO_4_, 7.4 mM HEPES.NaOH, 1.2 mM KH_2_PO_4_, 0.8 mM K_2_HPO_4_, pH 7.2) supplemented with 2% fetal calf serum (FCS) and gently pipetted to create a single-cell suspension. Cell suspensions were centrifuged at 1500 g, 5 min at 4 °C. Supernatant was then aspirated and the pellet was resuspended in 3 ml of BSS 2% FCS. Cells were filtered using a 100 μm cell strainer. Spleen cell suspensions were prepared by physical dissociation in 3 ml of BSS 2% FCS. Subsequently, 7 ml of Red Cell Removal Buffer (156 mM NH_4_Cl, 0.1 mM EDTA, and 12 mM NaHCO_3_) was added for 1 min at room temperature. After centrifugation the supernatants were aspirated and the pellet was resuspended in 5 ml of BSS 2% FCS. Cell suspensions were filtered using a 100 μm cell strainer. B-cell populations were defined as ProB-PreB1 (B220^+^ cKit^+^), PreB2 (B220^+^ cKit^−^ IgD^–^ IgM^−^), Immature (B220^+^ cKit^−^ IgD^−^ IgM^+^), Mature (B220^+^ cKit^−^ IgD^+^ IgM^+^). LSK (Lin^−^ Sca1^+^cKit^+^), LTHSC (Lin^−^ Sca-1^+^ cKit^+^ CD34^low/−^), STHSC (Lin^−^ Sca-1^+^ cKit^+^ CD34^+^ FLT3^−^), LMPP (Lin^−^ Sca-1^+^ CD34^+^ FLT3^+^), CLP (Lin^−^ Sca-1^int^ cKit^int^ IL7R^+^ FLT3^+^), ALP (Lin^−^ Sca-1^int^ cKit^int^ IL7R^+^ FLT3^+^ Ly6D^−^), BLP (Lin^−^ Sca-1^int^ cKit^int^ IL7R^+^ FLT3^+^ Ly6D^+^). Data was acquired using a LSRFortessa flow cytometer (BD, Franklin Lakes, NY, USA), and data analysis was performed using FlowJo software 10.3.0 (Treestar Inc, Ashland, USA).

### LSK gene expression analysis

Gene set tests were performed on microarray data generated from sorted LSK cells from adult wild-type C57BL/6, *Mpl* ^−/−^ and *Tpo* ^*Tg*^ mice. Total RNA was hybridized to Illumina MouseWG-6v2 bead chip arrays^15^. The microarray data are available at Array express (www.ebi.ac.uk/arrayexpress) under accession no. E-MTAB-2389. Results were normalized using the neqc(x) function in limma and gene expression was fitted to the linear model using empiric Bayes and array weights. Gene set tests were performed using gene sets obtained for the CLP, ProB and PreB populations from the Hemopedia atlas (haemosphere.org)^18^ using the top 77 upregulated genes for each population compared to the highest gene expression across the entire dataset. Using the Roast function in Limma, a self-contained rotational gene set testing method^37^, gene set enrichment was tested in *Mpl* ^−/−^ and *Tpo* ^*Tg*^ LSKs when compared to wild-type and leading-edge genes contributing to the enriched CLP expression signature in *Mpl* ^−/−^ LSK cells, as well as downregulated CLP expression signature in *Tpo* ^*Tg*^ LSK cells, were extracted (Supplementary Table S1).

### Analysis of B-lymphoid cell cycling

Single-cell suspensions from bone marrow and spleen of 4–5 week old mice were harvested as described above. Nucleated cell count was performed using trypan blue exclusion and 20 × 10^6^ cells were stained with antibodies to B220 (clone RA3-6B2; BD Pharmingen Franklin Lakes NY USA), CD19 (clone 1D3; ebiosciences San Diego CA USA), c-Kit (clone ACK4; WEHI mAb Facility, Parkville VIC Australia), IgD (clone 11–26 c.2a; Biolegend San Diego CA USA), and IgM (clone 5.1; WEHI mAb Facility) for 30 min on ice. Cells were then washed with BSS 2% FCS and stained with the appropriate secondary streptavidin-conjugated fluorochromes for another 30 min on ice. Subsequently, cells were fixed and permeabilized with Cytofix/Cytoperm (BD) according to the manufacturer’s instruction for 30 min on ice. Cells were then incubated with either FITC-conjugated anti-K_i_-67 antibody or the appropriate FITC-conjugated isotype control (BD) overnight at 4 °C. Lastly, 4′,6-diamidino-2-phenylindole (DAPI) (10 μg/ml) (Sigma-Aldrich, St. Louis, MO, USA) was added for 30 min at room temperature for analysis of DNA content. Cells were subsequently washed and resuspended with BSS 2% FCS and filtered using 40 μm cell strainer prior to analysis. Data was acquired using a LSR II flow cytometer (BD), and data analysis was performed using FlowJo software. Here, B-cell subsets were defined as: ProB-PreB1 (B220^+^ cKit^+^), PreB2 (B220^+^ CD19^+^ IgD^–^ IgM^−^), Immature (B220^+^ CD19^+^ IgD^−^ IgM^+^) and Mature (B220^+^ CD19^+^ IgD^+^ IgM^+^).

### Romiplostim

At 6–8 days old, Eμ-*myc* mice and their wild-type littermates were injected subcutaneously with 100 μg/kg Romiplostim^38^ (Nplate®, Amgen, Thousand Oaks, CA, USA) or saline vehicle. Injections were repeated every 3 days until mice were 5-weeks old.

### Statistics

Statistical significance between two treatment groups was analyzed using an unpaired Student’s *t* test with two-tailed *p-*values. One-way ANOVA with multiple comparison test was applied where appropriate (GraphPad Prism Version 7). *p < 0.05; **p < 0.005; ***p < 0.001 or as otherwise stated. Data are presented as mean ± SD or SEM, indicated in figure legends.

### Data availability statement

Figure 1 and Tables S1 and S2 have an associated data source. The microarray data are available at Array express (www.ebi.ac.uk/arrayexpress) under accession no. E-MTAB-2389.

## Electronic supplementary material

Supplementary information
