# Supplementary material for: Altered B-lymphopoiesis in mice with deregulated thrombopoietin signaling
Source: Sci Rep. 2017 Nov 2;7:14953. doi: 10.1038/s41598-017-15023-2 (PMC5668349; doi:10.1038/s41598-017-15023-2)
Supplement: Supplementary file 1 — Supplementary information [file 41598_2017_15023_MOESM1_ESM.pdf]

SUPPLEMENTARY INFORMATION

Altered B-lymphopoiesis in mice with deregulated thrombopoietin signaling.

Amanda E. Au[1,2], Marion Lebois[1], Starling A. Sim[1,2], Ping Cannon[1], Jason Corbin[1], Pradnya Gangatirkar[1], Craig D. Hyland[1], Diane Moujalled[1], Angelika Rutgersson[1,3], Fatme Yassinson[1,3], Benjamin T. Kile[1,2], Kylie D. Mason[4], Ashley P. Ng[1,2], Warren S. Alexander[1,2] and Emma C. Josefsson[1,2*]

SUPPLEMENTARY METHODS

Limiting dilution assay

CLPs (Lin$^-$ Sca-1^{int} cKit^{int} IL7Rα^+ FLT3$^+$), were sorted from lineage depleted C57BL/6 WT, *Mpl*$^{-/-}$ and *Tpo*^{Tg} bone marrow on a FACSAria cell sorter (BD) and seeded onto OP9 cells in the presence of OP9 media (α-MEM, 10% FCS, 100 U/ml penicillin, 100 μg/ml streptomycin, 1 mM L-glutamine, 10 mM HEPES, 1 mM sodium pyruvate (GIBCO BRL), and 50 μM β-mercaptoethanol (Sigma-Aldrich) supplemented with IL-7 10 ng/ml (Peprotech Rocky Hill, NJ USA), Flt3L 500 ng/ml and SCF 100ng/ml. Serial dilutions were performed in 96 well plates, starting with 100 cells/well in row 1 and serially diluting it down 8 times to 1.25 cells per well [1]. The lineage cocktail contained biotinylated anti-CD2 (clone RM2.1), CD3 (clone KT3.1.1), CD4 (clone GK1.5), CD5 (clone 53-7.3), CD8 (clone 53.6.7), CD19 (clone 1D3), B220 (clone RA3-6B2), GR1 (clone RB6-8C5), Ly6G (clone 1A8), Ter119 (clone Ly76), CD11b (clone M1/70), NK1.1 (PK136) and F4/80 (F480) WEHI mAb Facility, Parkville VIC Australia. The lineage positive bone marrow cells were removed with magnetic anti-biotin beads adhering to a column (Miltenyi Biotec). Analysis was performed according to ELDA (http://bioinf.wehi.edu.au/software/elda/) [2].

Mpl receptor expression

B-cell subsets in bone marrow and spleen cells, from 6 week old mice, were stained with the antibodies B220-APCCy7, IgD-PE, IgM-Alexa647, cKit-PerCP5.5, and biotinylated rat-anti-mouse Mpl (clone AMM2; Immuno-Biological Laboratories, Minneapolis, MN, USA) for 30 min on ice. LSK and CLP bone marrow cells were stained with Alexa700 conjugated anti- CD2, CD3, CD4, CD5, CD8, CD19, B220, GR1, Ly6G, Ter119, CD11b, NK1.1 and F4/80 (WEHI mAb Facility) as well as Sca-1-Alexa594 (clone D7 Ly6A/E; WEHI mAb Facility), CKit-PerCPCy5.5 (clone ACK4; ebiosciences), Flt3-PE (clone A2F10; Biolegend San Diego CA USA), IL7r-APC (clone A7R34; Biolegend) and biotinylated rat-anti-mouse Mpl (clone AMM2) for 30 min on ice. The cells were then washed and stained with Streptavidin PE-Cy7 for 30 min

on ice. Subsequently, the cells were washed and samples acquired on a LSRFortessa (BD) flow cytometer. Live cells were defined as fluorogold negative. Isotype negative control Biotin mouse IgG1k (clone MOPC-21; Biolegend).

Histology

Lymph nodes and sternums were collected in 10% formalin and H&E sections prepared for pathological analysis. Images were acquired on a Nikon Eclipse E600 microscope equipped with AxioCam MRc5 (Zeiss, Oberkochen, Germany) and AxioVision 4.8.

Analysis of apoptosis

Single-cell suspensions were prepared from bone marrow of 4-5 week-old female mice. Cells were centrifuged and resuspended in 10 ml of Dulbecco's Modified Eagles Medium (DMEM; 0.1% (w/v) D-glucose, 0.011% (w/v) sodium pyruvate, 0.034% (w/v) $NaHCO_3$, 100 μg/ml streptomycin and 100 IU/ml penicillin) supplemented with 10% FCS, 50 μM β-mercaptoethanol, and 250 μM L-asparagine (Sigma-Aldrich). Subsequently, a nucleated cell count was performed using trypan blue exclusion and the cell concentration was adjusted to 2×10^6 cells/ml. Equal numbers of cells, 8×10^6 cells were cultured in wells of a tissue cultured -treated 6-well plate in 37°C incubator with 10% CO_2. Cells were analyzed at defined time points post culture. Cells were centrifuged and supernatant was aspirated. Pellets were resuspended in BSS 2% FCS supplemented with 1 mM $CaCl_2$ and incubated with antibodies for surface markers: B220, CD19, c-Kit, IgD, and IgM for 30 min on ice. Cells were washed with BSS 2% FCS 1mM $CaCl_2$ and stained with the appropriate streptavidin-conjugated fluorochromes and Annexin V (BD Biosciences (Franklin Lakes NY, USA) for another 30 min on ice. Lastly, cells were washed with BSS 2% FCS 1 mM $CaCl_2$ and stained with fluorogold prior to analysis. Samples were run on the LSR II flow cytometer (BD) and data analysis was performed using FlowJo software. Apoptotic (Annexin V^+) B-cell subsets were defined as PreB2

($B220^{+}$ $CD19^{+}$ c-Kit^{-} IgD^{-} IgM^{-}), Immature ($B220^{+}$ $CD19^{+}$ c-Kit^{-} IgD^{-} IgM^{+}), Mature ($B220^{+}$ $CD19^{+}$ c-Kit^{-} IgD^{+} IgM^{+}) and total B-cells ($B220^{+}$ $CD19^{+}$).

Analysis of HSC and progenitor cell cycling

Single-cell suspensions were prepared from bone marrow of 4-5 week-old female mice. Nucleated bone marrow cell count was performed using trypan blue exclusion and 20×10^{6} – 40×10^{6} cells were stained with PE-conjugated antibodies against mature hematopoietic cell markers: B220, CD19, CD4, CD8, Gr-1, Ter-119, F4/80, and Ly6G WEHI mAb Facility as well as Sca-1 and c-Kit. Subsequently, cells were fixed and permeabilised with Cytofix/Cytoperm (BD) according to the manufacturer's instruction for 30 min on ice. Cells were then incubated with either FITC-conjugated anti-K_i-67 antibody or the appropriate FITC-conjugated isotype control (BD pharmingen) overnight at 4°C. Lastly, 4',6-diamidino-2-phenylindole (DAPI) (10 µg/ml) (Sigma-Aldrich) was added for 30 min at room temperature for analysis of DNA content. Cells were subsequently washed and resuspended with BSS 2% FCS and filtered using 40 µm cell strainer (BD) prior to analysis. Samples were run on a LSR II flow cytometer, and data analysis was performed using FlowJo software. HSC and progenitor cells were defined in Lineage-negative (Lin-) Sca-1^{+} c-Kit^{+} (LSK) population.

REFERENCES

1. Greig KT, de Graaf CA, Murphy JM, Carpinelli MR, Pang SH, Frampton J, *et al.* Critical roles for c-Myb in lymphoid priming and early B-cell development. *Blood* 2010 Apr 8; **115**(14)**:** 2796-2805.

2. Hu Y, Smyth GK. ELDA: extreme limiting dilution analysis for comparing depleted and enriched populations in stem cell and other assays. *Journal of immunological methods* 2009 Aug 15; **347**(1-2)**:** 70-78.

SUPPLEMENTARY TABLES

Table S1.
CLP signature genes upregulated or downregulated in *Mpl*$^{-/-}$ and *Tpo*^{Tg} LSKs, respectively.

Table S2.
CLP, ProB and PreB gene expression signatures in *Mpl*$^{-/-}$ and *Tpo*^{Tg} LSKs.

SUPPLEMENTARY FIGURES

Figure S1. (**a**) Hematopoietic stem and progenitor cells per femur in 4-5 week old mice. WT (n=8), *Mpl*$^{-/-}$ (n=6) and *Tpo*^{Tg} (n=7). Mean ± SEM. Statistical significance was generated by Student's unpaired t-test. *$p < 0.05$. Gating strategies for (**b**) LSKs and CLPs and (**c**) ProB-PreB1, PreB2, immature, and mature B-cells.
(**d**) Mpl surface expression was assessed by flow cytometry using wild-type bone marrow for LSK and CLP subsets, while B-cell subsets were analysed using wild-type bone marrow and spleen from 6 week-old mice. Cells obtained from *Mpl*$^{-/-}$ mice were included as a negative control. Negative isotype control: Biotin mouse IgG1k. Representative histogram derived from a single experiment is depicted.

Figure S2. Limiting dilution assay using Extreme Limiting Dilution Analysis (ELDA) of B-cell clonogenicity of CLPs sorted from wild-type, *Mpl*$^{-/-}$ and *Tpo*^{Tg} mice. The data includes 3 separate experiments.

Figure S3. Preneoplastic Eμ-*myc* cells do not express Mpl. Mpl protein surface expression was assessed by flow cytometry in B-cell subsets from bone marrow and spleen of 4-5 week old preneoplastic Eμ-*myc* mice. Histograms are derived from 1 representative mouse. Negative control: inclusion of fluorescently conjugated Streptavidin in the absence of biotinylated rat-anti-mouse Mpl.

Figure S4. Spleen cellularity and lymph node weight in terminally ill mice.
(**a**) Spleen cellularity was determined by flow cytometry and includes Eμ-*myc* (n=22), *Mpl*$^{-/-}$ Eμ-*myc* (n=16), *Tpo*^{Tg} Eμ-*myc* (n=13) terminally ill mice. (**b**) Lymph node (axillary, brachial, inguinal and mesenteric) weights in terminally ill Eμ-*myc* (n=23), *Mpl*$^{-/-}$ Eμ-*myc* (n=47), *Tpo*^{Tg} Eμ-*myc* (n=44) mice. Graphs show mean ± SEM. Statistical significance was generated by Student's unpaired t-test. *$p < 0.05$

Figure S5. Bone marrow histology of healthy and terminally ill mice.
H&E stained sternal sections from healthy wild-type (WT), *Mpl*$^{-/-}$ and *Tpo*^{Tg} mice and

terminally ill Eμ-*myc* mice. Extravasation is indicated by asterisks. Images were acquired on a Nikon Eclipse E600 microscope equipped with AxioCam MRc5 and AxioVision 4.8. (**a**) Scale bar: 50 μm, (**b**) Scale bar: 10 μm

Figure S6. Lymph node histology of terminally ill mice.

H&E stained sections of lymph nodes from terminally ill Eμ-*myc* mice and a healthy wild-type (WT) mouse. Sections from 3 mice per genotype are displayed and indicated by #1-3. Asterisks (yellow) indicate areas of hemorrhage. BV = blood vessel. Images were acquired on a Nikon Eclipse E600 microscope equipped with AxioCam MRc5 and AxioVision 4.8. (**a**) Scale bar: 50 μm, (**b**) Scale bar: 10 μm.

Figure S7. Apoptosis is unaffected in cultured bone marrow B-cells subsets of preneoplastic *Mpl*$^{-/-}$Eμ-*myc* and *Tpo*^{Tg} Eμ-*myc* mice.

In vitro cell survival assay was performed on bone marrow cells from WT (n=12), *Mpl*$^{-/-}$ (n=7), *Tpo*^{Tg} (n=8), Eμ-*myc* (n=10), *Mpl*$^{-/-}$Eμ-*myc* (n=10) and *Tpo*^{Tg} Eμ-*myc* 4-5 week-old female mice (n=3). Bone marrow cells were cultured under conditions of cytokine deprivation and acquired and stained at defined time points. Apoptosis was defined in B-cell subsets by the expression of Annexin-V. Two-way analysis of variance (ANOVA), followed by Tukey's post-hoc test for individual pair-wise comparison was used to determine statistical significance. Graphs show mean ± SD. **= p<0.005 and ***=p<0.0001

Figure S8. Eμ-*myc* LSK analysis.

(**a**) Mpl surface expression was assessed by flow cytometry using wild-type and Eμ-*myc* bone marrow for LSK and CLP subsets from 6 week-old mice. Cells obtained from *Mpl*$^{-/-}$ and *Mpl*$^{-/-}$ Eμ-*myc* mice were included as a negative controls. Negative isotype control: Biotin mouse IgG1k. Representative histogram derived from a single experiment is depicted. (**b**) Flow cytometric analyses of bone marrow from WT (n=12), *Mpl*$^{-/-}$ (n=13), *Tpo*^{Tg} (n=7), Eμ-*myc* (n=10), *Mpl*$^{-/-}$ Eμ-*myc* (n=9), and *Tpo*^{Tg} Eμ-*myc* (n=7) mice showing percentages of LSKs in bone marrow. (**c**) Cell cycle status of bone marrow LSKs, enriched for hematopoietic stem cells and

progenitors (HSPCs). Mean percentages of bone marrow LSK cells that are in G0, G1, or S, G2, M phase of the cell cycles determined by K_i-67/DAPI staining. WT (n=17), *Mpl*$^{-/-}$ (n=14), *Tpo*^{Tg} (n=10), Eμ-*myc* (n=11), *Mpl*$^{-/-}$ Eμ-*myc* (n=9), and *Tpo*^{Tg} Eμ-*myc* (n=7) mice. Mice were 4-5 week-old female mice. Graphs show mean ± SEM. Statistical significance was generated by Student's unpaired t-test. *$p < 0.05$; **$p < 0.005$; ***$p < 0.001$.

Table S1

CLP signature Leading Edge Probes upregulated in *Mpl-/-* LSKs				
**ProbeID**	**Gene**	**lfc**	**p**	**t**
3180468	Slc27a2	1.22335972	6.32E-09	9.45457471
130131	Dntt	1.79970448	2.70E-08	8.663485951
2470356	Cox6a2	1.72640972	3.04E-05	5.320272307
5820672	Tcp1	0.99512369	0.00018675	4.542717912
5130075	Sdc1	0.6042297	0.00020331	4.506700231
2510019	Ddx4	1.09479804	0.00042721	4.192659569
7570544	Ctr9	0.73920401	0.00045534	4.165712654
1110136	Cd244	0.80576312	0.00078182	3.937247829
6660402	Speer6-ps1	0.59481883	0.00102141	3.824086308
1340220	Rhbdl3	0.59899857	0.00105653	3.809761706
270301	H2afy2	0.57295076	0.00106532	3.806249217
6130484	Elk3	0.57622966	0.00123353	3.744073483
4570193	Tcam1	0.74476894	0.00173096	3.600017642
3800537	Ttc13	0.42372775	0.00182937	3.576448382
730528	Phax	0.39222744	0.00323039	3.332783867
6760343	Trim27	0.38949798	0.00354453	3.292748808
6940482	Mtdh	0.56146043	0.00367892	3.276670487
5810246	Dnajc2	0.50904161	0.00408513	3.231337809
240593	Mdh1	0.52779663	0.00445984	3.193256729
1110273	Nap1l1	0.36258561	0.00483958	3.15771463
6450594	Ppm1f	0.43887234	0.00554216	3.098562956
4540064	Cd244	0.52603923	0.00708209	2.990908984
4200482	Gphn	0.37188096	0.00866625	2.901550855
7150491	Trim27	0.35468982	0.0100647	2.834855653
4230376	Mtdh	0.33695987	0.0115894	2.771562588
3830241	Ephb2	0.51811427	0.01263664	2.732535399
6940487	Phf14	0.29107553	0.0192126	2.540974698
4260014	Them7	0.44914375	0.01990452	2.524583615
580259	Ctnnd2	0.47730826	0.02147067	2.489370169
7510392	Coil	0.36949496	0.02519837	2.414355641
620711	Dnajc2	0.50753899	0.03028214	2.327181465
5810767	Sdc1	0.61278634	0.03131293	2.311172804
7150392	Btbd3	0.47135553	0.03714996	2.228745011
5690273	Ephb2	0.47206356	0.04036369	2.188303149
2070468	Il12a	0.38669071	0.04212811	2.167331223
6450309	Slc27a2	0.36709981	0.04316642	2.155360026
4730164	Irf2bp2	0.33161408	0.04876056	2.095029974
2940689	Nap1l1	0.45192469	0.04950118	2.087517241
780204	Xpc	0.23290649	0.04953793	2.087147117
6550097	Egln1	0.51288001	0.05111966	2.071445491
4200626	Esr1	0.49422449	0.07272987	1.891648872
6290471	Esr1	0.33089166	0.07831118	1.853006601
3370612	Egln1	0.33341244	0.07860792	1.851020357
5890255	Cd93	0.23678539	0.08875798	1.786727319
3290008	Sh3rf1	0.3056014	0.09417465	1.754986504
7330722	Cdk19	0.22742462	0.11290307	1.656120798

CLP signature Leading Edge Probes downregulated in *TpoTg* LSKs

ProbeID	Gene	lfc	p	t
3180468	Slc27a2	-0.98483201	1.11E-07	-7.92872412
1340220	Rhbdl3	-1.09615584	4.29E-07	-7.26269254
2070468	Il12a	-1.06358383	4.08E-06	-6.209928624
6450022	Notch1	-0.82155381	4.58E-06	-6.156871044
6450309	Slc27a2	-0.88972314	2.30E-05	-5.441816279
6250669	Enkd1	-0.87315034	8.24E-05	-4.890612937
3830241	Ephb2	-0.84444105	0.000148688	-4.639411622
1770343	Rgs8	-0.64045114	0.000456214	-4.164906342
130131	Dntt	-0.78703271	0.000764482	-3.946731593
2510019	Ddx4	-0.96366945	0.000973415	-3.84447473
1030605	Itpk1	-0.43092322	0.001831523	-3.575947088
4260014	Them7	-0.56531329	0.003404529	-3.310144325
5810347	Phf14	-0.39796623	0.0043013	-3.208974082
540544	Pgls	-0.38182347	0.004555714	-3.184013496
7560543	Hoxa9	-0.63240067	0.005126454	-3.132615883
4490017	Tap2	-0.47858979	0.006186133	-3.050408732
1570014	Msi2	-0.47900726	0.006688703	-3.016083144
4640072	Tcam1	-0.55609695	0.007342089	-2.974999673
3460754	Them7	-0.43213264	0.007396091	-2.97176343
2470356	Cox6a2	-0.85249897	0.012519001	-2.736762954
6250754	Ylpm1	-0.32784207	0.024005902	-2.437158608
2030132	Trim27	-0.66666296	0.030763354	-2.319646492
5860093	Gpc2	-0.55638426	0.03078143	-2.319365567
3120039	Cacnb2	-0.42310537	0.037361494	-2.225986734
4780681	Cdk19	-0.24436355	0.040463489	-2.187094931
4540064	Cd244	-0.35237037	0.049545225	-2.087073629
2260228	Tcam1	-0.36960306	0.058991756	-1.999247117
5270563	Tcof1	-0.27262592	0.081133231	-1.83437742
3800537	Ttc13	-0.20396241	0.087660055	-1.793364319
5810327	Btbd3	-0.29744144	0.101634097	-1.713754281
780427	Egln1	-0.28580664	0.114882181	-1.646507682
770450	Msi2	-0.2633858	0.123437255	-1.606495014

Table S2

Mpl-/- vs WT LSK	NGenes	PropDown	PropUp	Direction	PValue	FDR	PValue.Mixed	FDR.Mixed
CLP	140	0.157142857	0.38571429	Up	1.00E-04	1.00E-04	1.00E-04	1.00E-04
ProB	71	0.126760563	0.29577465	Up	1.00E-04	1.00E-04	0.00029997	0.00029997
PreB	91	0.142857143	0.18681319	Up	0.00029997	0.00037496	0.00079992	0.00079992

TpoTg vs WT LSK	NGenes	PropDown	PropUp	Direction	PValue	FDR	PValue.Mixed	FDR.Mixed
CLP	140	0.271428571	0.11428571	Down	1.00E-04	1.00E-04	1.00E-04	1.00E-04
ProB	71	0.295774648	0.09859155	Down	1.00E-04	1.00E-04	1.00E-04	1.00E-04
PreB	91	0.175824176	0.14285714	Down	0.00319968	0.00377962	0.00019998	0.00019998

Figure S1

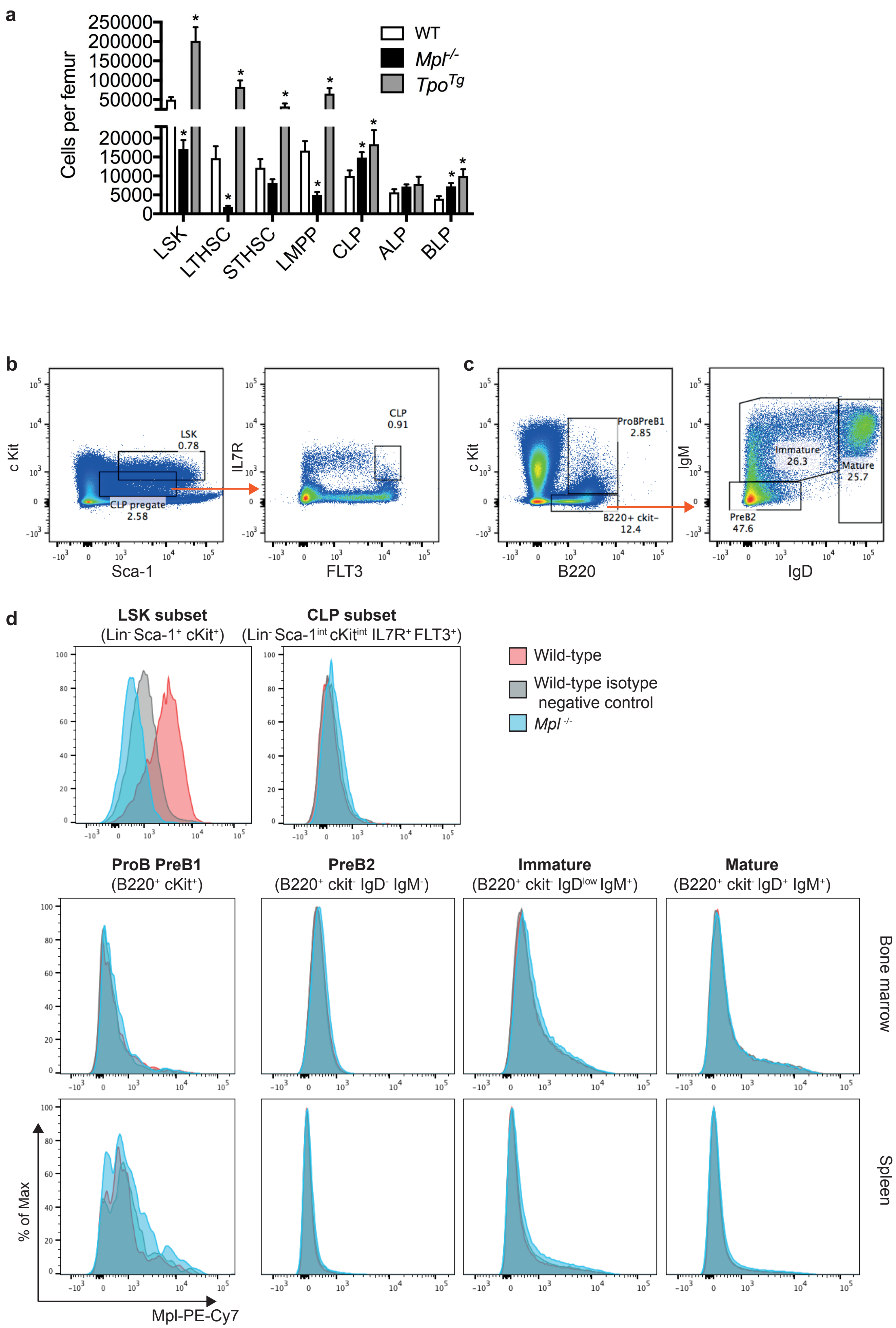
a
Cells per femur
250000
200000
150000
100000
50000
20000
15000
10000
5000
0
WT
Mpl-/-
TpoTg
LSK
LTHSC
STHSC
LMPP
CLP
ALP
BLP
b
c Kit
Sca-1
LSK
0.78
CLP pregate
2.58
IL7R
FLT3
CLP
0.91
c
c Kit
B220
ProBPreB1
2.85
B220+ ckit-
12.4
IgM
IgD
Immature
26.3
Mature
25.7
PreB2
47.6
d
LSK subset
(Lin- Sca-1+ cKit+)
CLP subset
(Lin- Sca-1int cKitint IL7R+ FLT3+)
Wild-type
Wild-type isotype negative control
Mpl -/-
ProB PreB1
(B220+ cKit+)
PreB2
(B220+ ckit- IgD- IgM-)
Immature
(B220+ ckit- IgDlow IgM+)
Mature
(B220+ ckit- IgD+ IgM+)
Bone marrow
Spleen
% of Max
Mpl-PE-Cy7

Figure S2

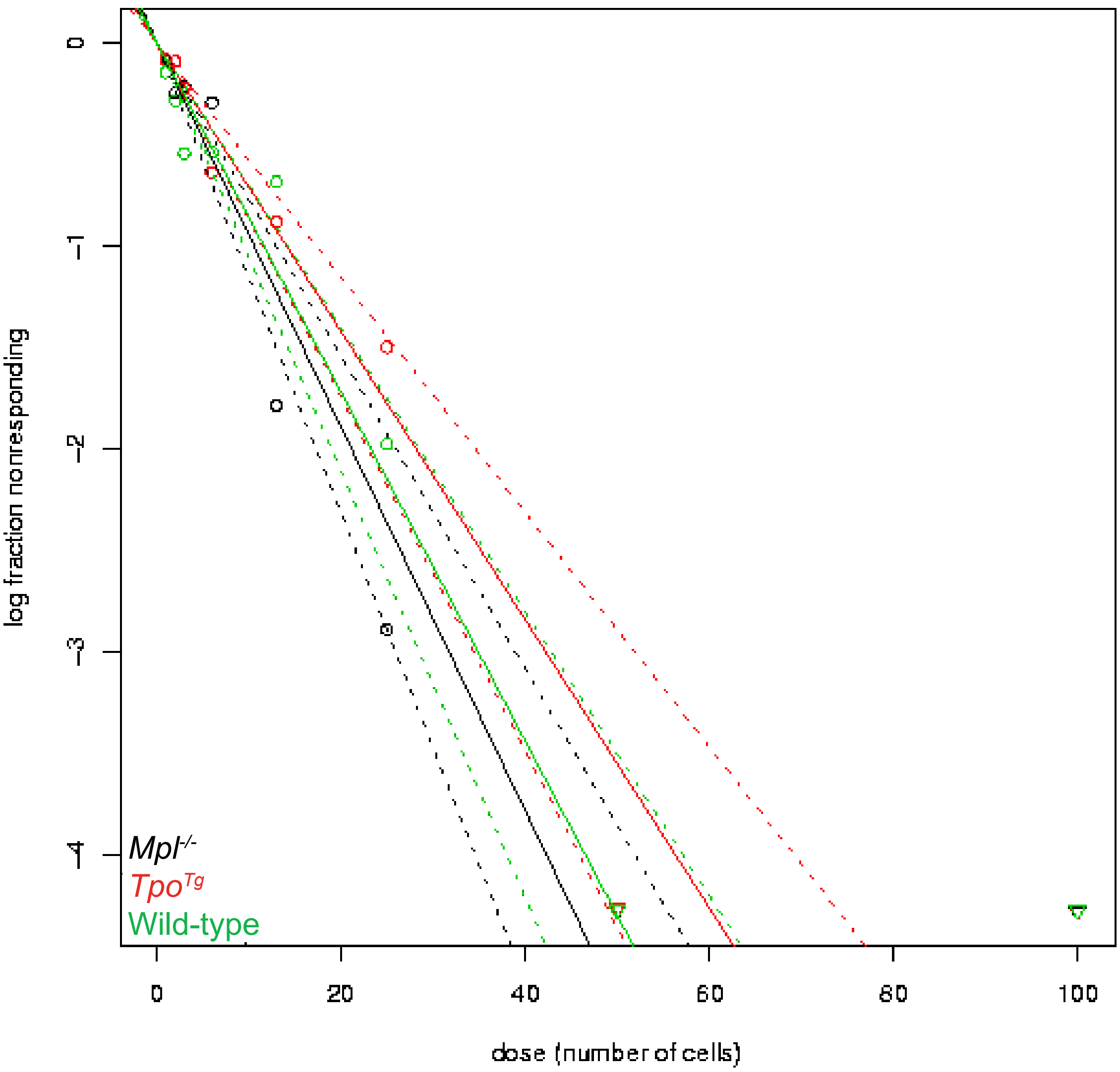

log fraction nonresponding
0
−1
−2
−3
−4
0
20
40
60
80
100
dose (number of cells)
Mpl-/-
*Tpo*Tg
Wild-type

Figure S3

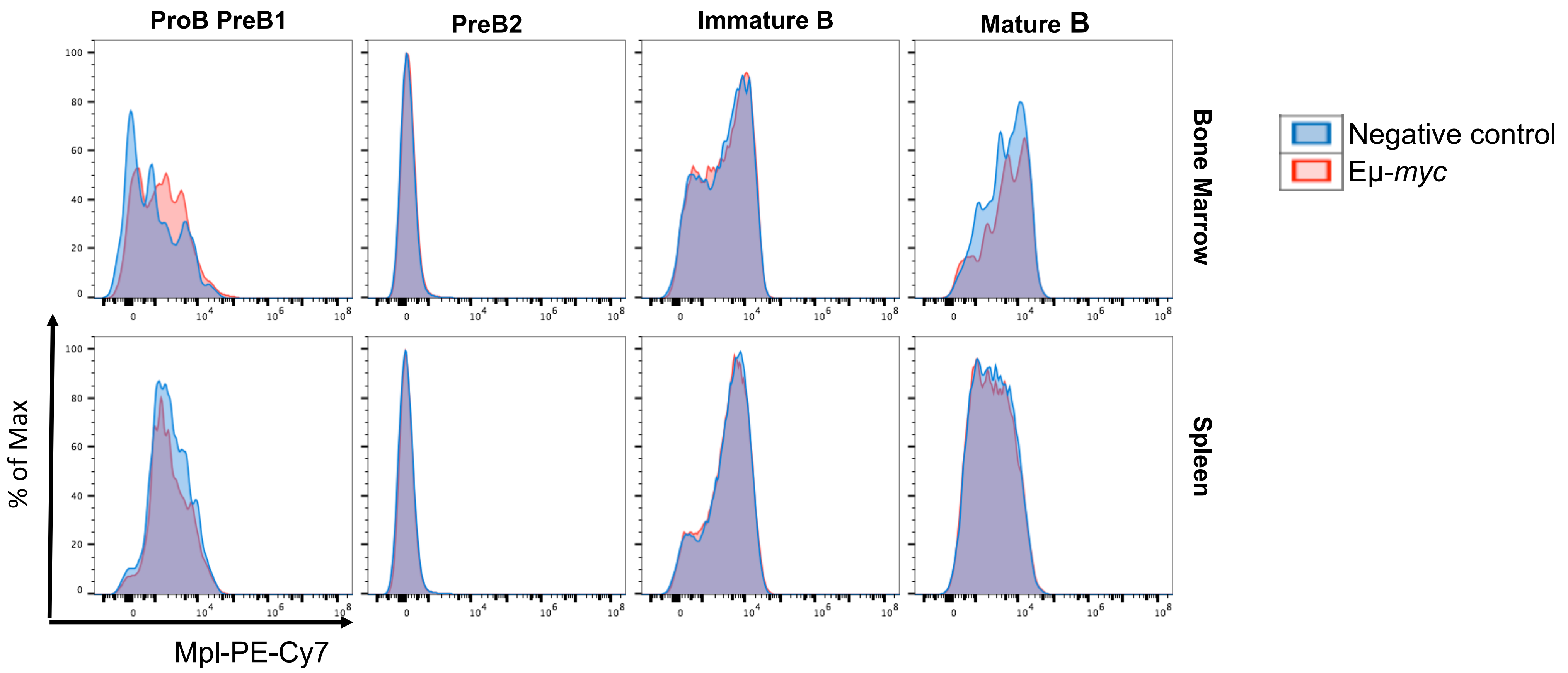
ProB PreB1
PreB2
Immature B
Mature B
Bone Marrow
Spleen
% of Max
Mpl-PE-Cy7
Negative control
Eμ-*myc*

Figure S4

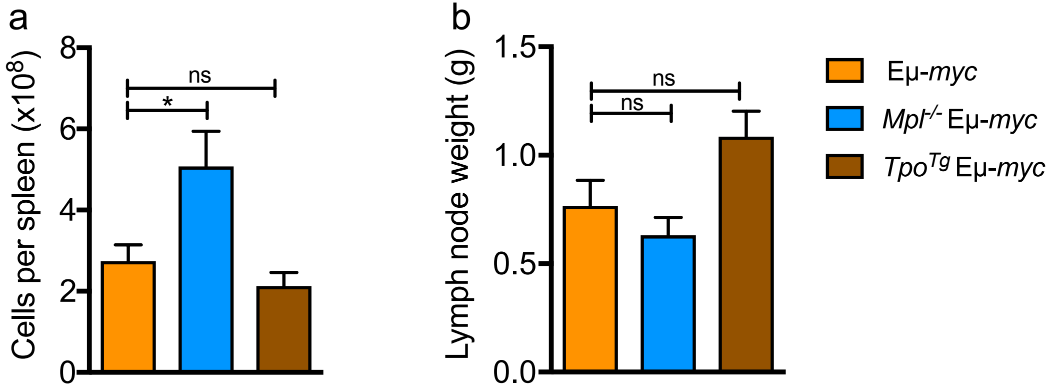

a
Cells per spleen (x10^8)
8
6
4
2
0
ns
*
b
Lymph node weight (g)
1.5
1.0
0.5
0.0
ns
ns
Eμ-myc
Mpl-/- Eμ-myc
TpoTg Eμ-myc

Figure S5a

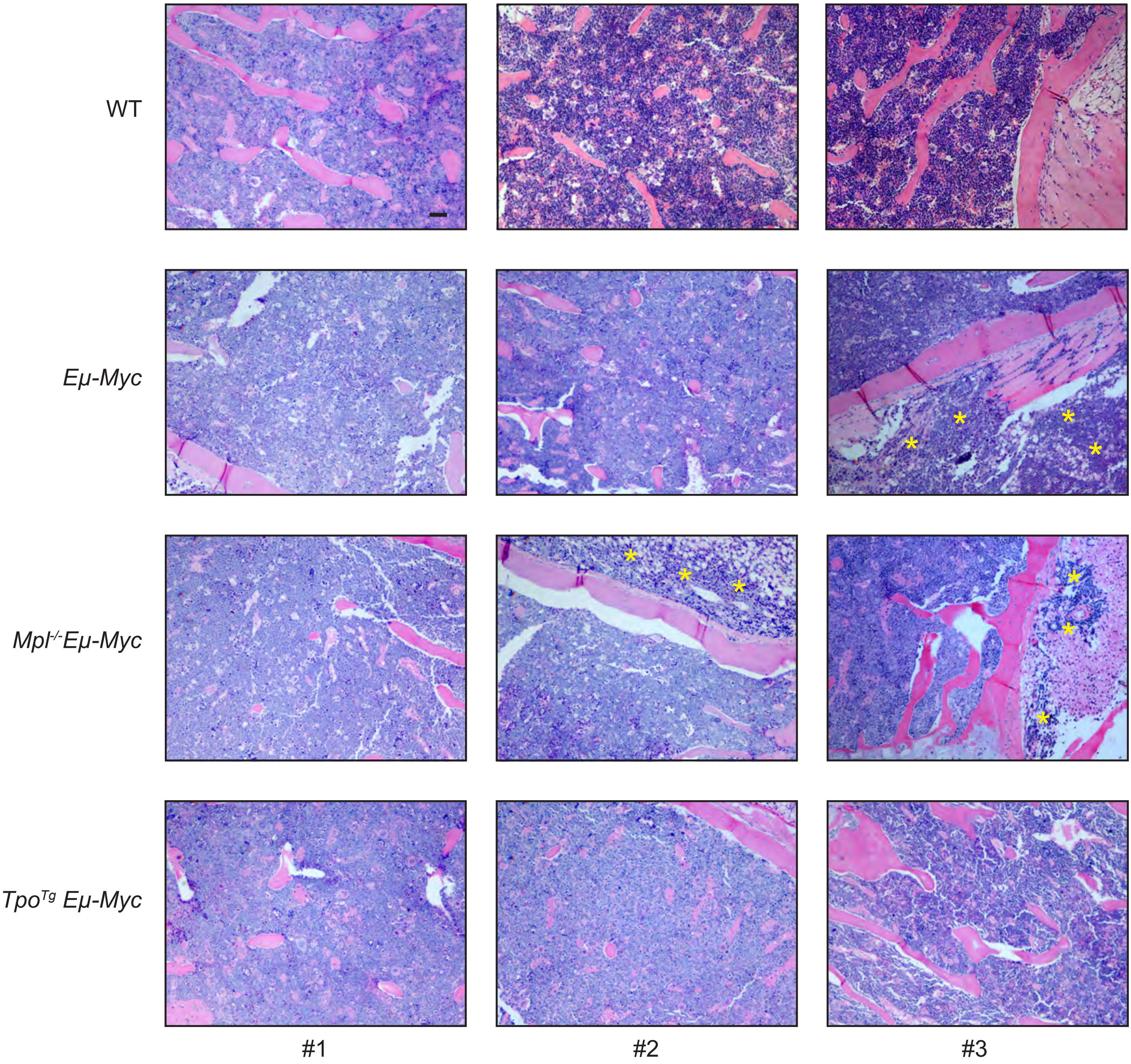

Figure S5b

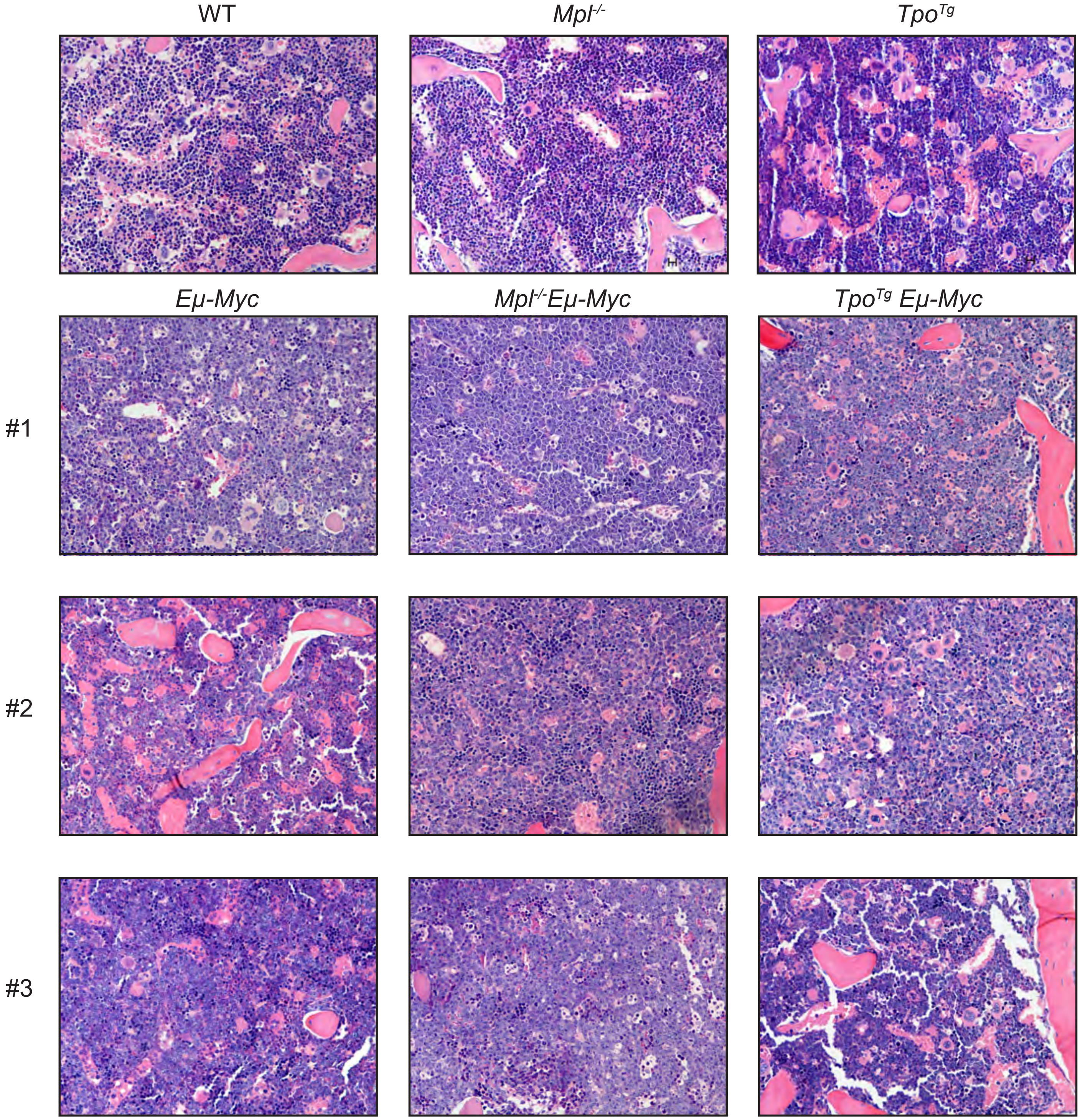

Figure S6a

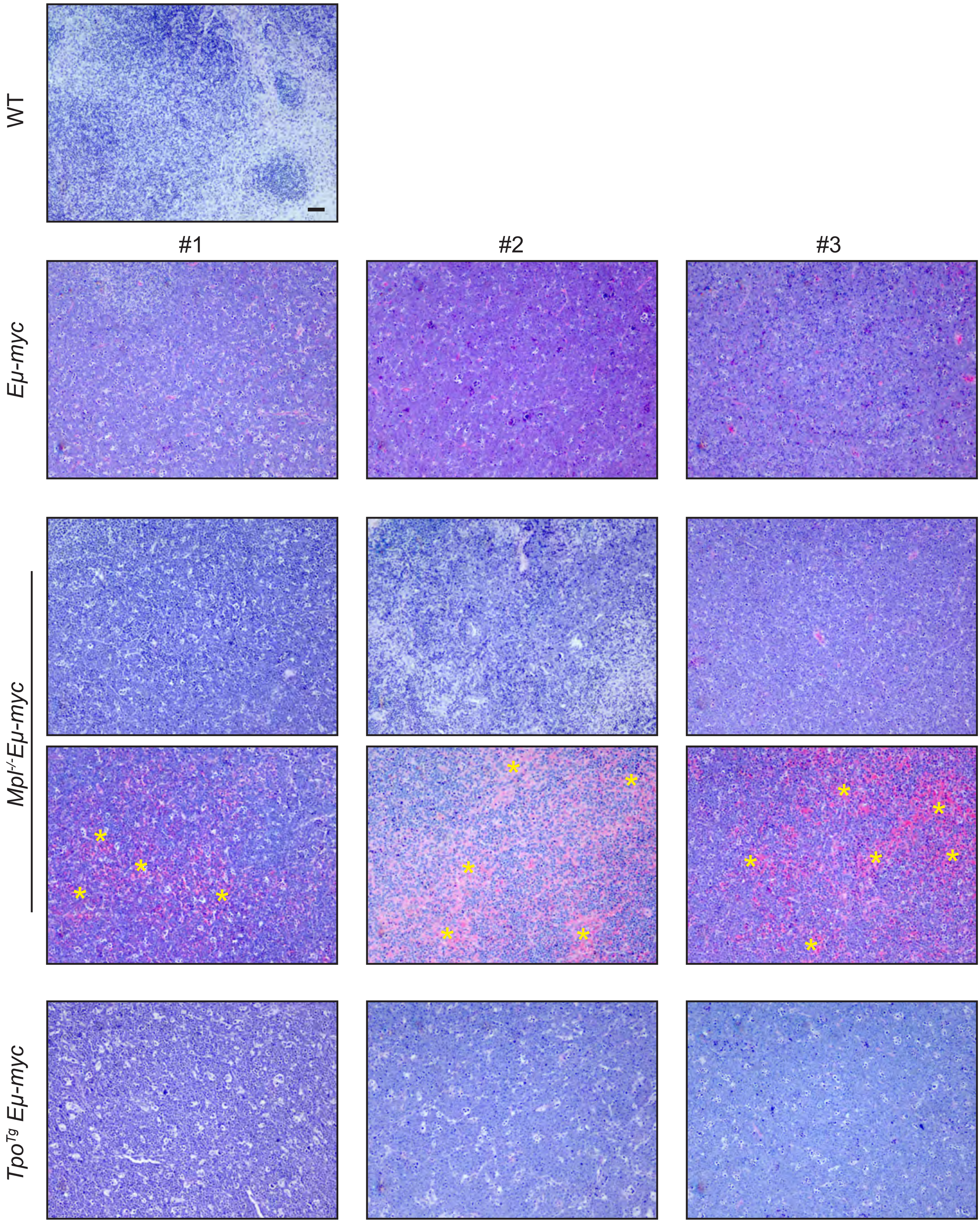

Figure S6b

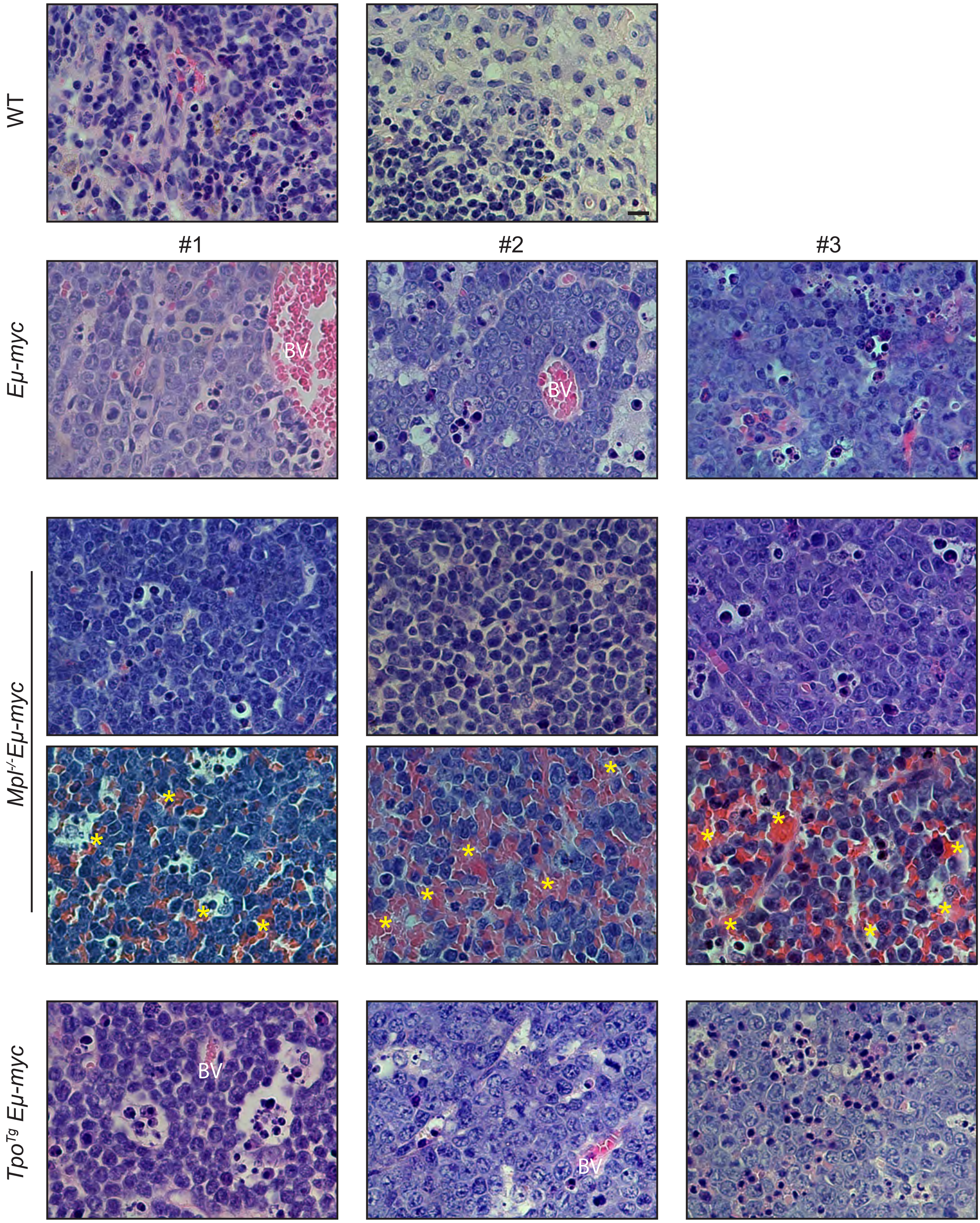

Figure S7

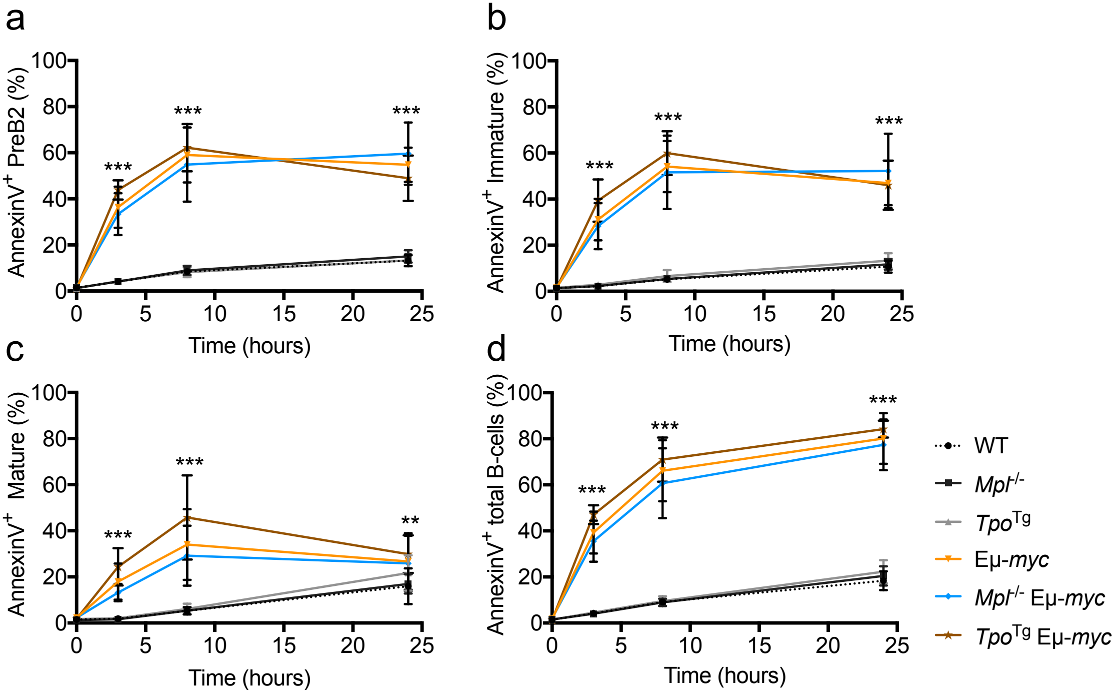

a
AnnexinV+ PreB2 (%)
Time (hours)
b
AnnexinV+ Immature (%)
Time (hours)
c
AnnexinV+ Mature (%)
Time (hours)
d
AnnexinV+ total B-cells (%)
Time (hours)
WT
Mpl-/-
TpoTg
Eμ-myc
Mpl-/- Eμ-myc
TpoTg Eμ-myc

Figure S8

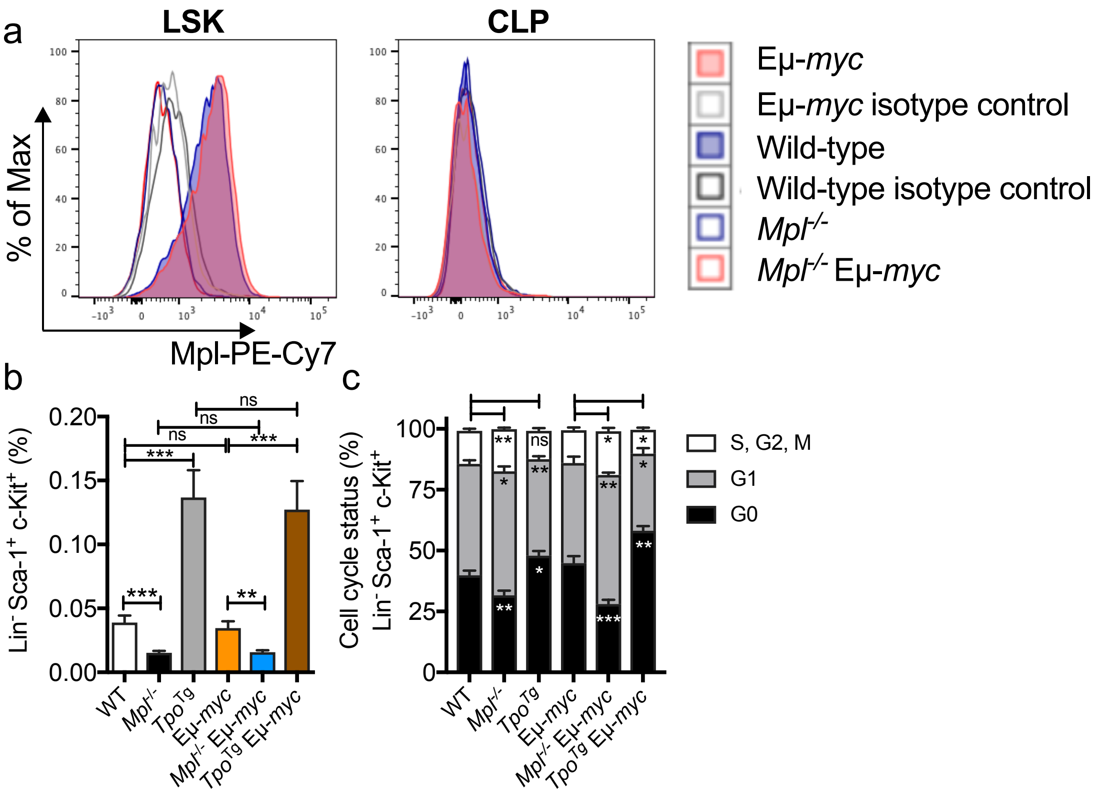
a
LSK
CLP
% of Max
Mpl-PE-Cy7
Eμ-myc
Eμ-myc isotype control
Wild-type
Wild-type isotype control
Mpl-/-
Mpl-/- Eμ-myc
b
Lin- Sca-1+ c-Kit+ (%)
WT
Mpl-/-
TpoTg
Eμ-myc
Mpl-/- Eμ-myc
TpoTg Eμ-myc
c
Cell cycle status (%)
Lin- Sca-1+ c-Kit+
S, G2, M
G1
G0
